# Comparison of deep learning‐based recurrence‐free survival with random survival forest and Cox proportional hazard models in Stage‐I NSCLC patients

**DOI:** 10.1002/cam4.6479

**Published:** 2023-08-29

**Authors:** İrem Kar, Gökhan Kocaman, Farrukh İbrahimov, Serkan Enön, Erdal Coşgun, Atilla Halil Elhan

**Affiliations:** ^1^ Department of Biostatistics Ankara University School of Medicine Ankara Turkey; ^2^ Department of Thoracic Surgery Ankara University School of Medicine Ankara Turkey; ^3^ Genomics Team, Microsoft Research & AI Redmond Washington USA

**Keywords:** Cox proportional hazard, DeepSurv, non‐small cell lung cancer, random survival forest

## Abstract

**Background:**

The curative treatment for Stage I non‐small cell lung cancer (NSCLC) is surgical resection. Even for Stage I patients, the probability of recurrence after curative treatment is around 20%.

**Methods:**

In this retrospective study, we included 268 operated Stage I NSCLC patients between January 2008 and June 2018 to analyze the prognostic factors (pathological stage, histological type, number of sampled mediastinal lymph node stations, type of resection, SUVmax of the lesion) that may affect relapse with three different methods, Cox proportional hazard (CoxPH), random survival forest (RSF), DeepSurv, and to compare the performance of these methods with Harrell's C‐index. The dataset was randomly split into two sets, training and test sets.

**Results:**

In the training set, DeepSurv showed the best performance among the three models, the C‐index of the training set was 0.832, followed by RSF (0.675) and CoxPH (0.672). In the test set, RSF showed the best performance among the three models, followed by DeepSurv with 0.677 and CoxPH methods with 0.625.

**Conclusion:**

In conclusion, machine‐learning techniques can be useful in predicting recurrence for lung cancer and guide clinicians both in choosing the adjuvant treatment options and best follow‐up programs.

## 1 INTRODUCTION

Lung cancer is the second most common type of cancer in the world and it ranks first in cancer‐related death rates. More than 2 million new cases are seen every year.^1^ Non‐small cell lung cancer (NSCLC) accounts for 85% of lung cancer.^2^ Increasing technical developments have made it possible to detect lung cancer in the early stages. In this way, more patients could get a chance for curative treatment. The curative treatment method for Stage I NSCLC is surgical resection.^3^ Even for Stage I patients, the probability of recurrence after curative treatment is around 20%. Adjuvant chemotherapy is not usually given in Stage I patients.^4^ If patients operated for Stage I NSCLC who are at risk for relapse could be predicted, better adjuvant treatment and follow‐up strategies can be developed. Statistical survival analysis methods and supervised machine learning models can be used to predict recurrence. Technique such as the Cox proportional hazard (CoxPH) model can provide a time‐based analysis of risk factors for the patient and is one of the most widely used techniques.^5^ Despite its popularity, the CoxPH model works with assumptions that are not always possible, such as proportional hazards, and it cannot provide an analysis of heterogeneous data (e.g., medical images, genetic sequences, etc.). These limitations are tried to be overcome with this machine learning methods, such as DeepSurv^6^ and random survival forest (RSF).^7^

There are some literature applying machine learning to various types of cancer datasets have reported good results. Kim et al,^8^ used the DeepSurv method for survival prediction in 255 oral squamous cell carcinoma patients and validated its performance. They also compared the survival prediction performances of DeepSurv, RSF and the CoxPH models. DeepSurv showed the best performance among the three models, the C‐index of the testing sets reaching 0.781, followed by RSF (0.764) and CoxPH (0.694), respectively. Liu et al,^9^ conducted a study to examine the prognostic significance and the potential role of treatment decisions based on pathological microscopic features in patients diagnosed with nasopharyngeal carcinoma (NPC). Employing the neural network DeepSurv, they meticulously analyzed the pathological microscopic features (DSPMF) of the patients. Subsequently, utilizing the time‐dependent receiver operating characteristic (ROC) analysis, they effectively categorized the patients into distinct high‐risk and low‐risk groups. Ultimately, the study's findings led them to conclude that the DSPMF serves as a reliable prognostic tool for assessing survival risk in patients with NPC and could potentially offer a valuable guidance for treatment decisions. Huang et al,^10^ investigated multiple deep learning‐based models, such as Cox‐nnet, DeepSurv, and their own method called AutoEncoder with Cox regression network (AECOX) across 12 cancer types from The Cancer Genome Atlas (TCGA). Utilizing the C‐index metric, they compared the prediction performance of these methods and observed that deep learning‐based algorithms outperformed traditional machine learning‐based models. As a result, the study highlighted the superior predictive capabilities of deep learning approaches in the context of cancer prognosis. Byun et al,^11^ compared the performance of RSF and DeepSurv models with the CoxPH model for predicting recurrence‐free survival and cancer‐specific survival in non‐metastatic clear cell RCC patients. The findings revealed that DeepSurv demonstrated superior predictive performance compared to both CoxPH and RSF models.

This study aimed to analyze the prognostic factors that may affect relapse in patients with operated Stage I NSCLC with three different methods such as CoxPH, RSF, and DeepSurv, and compare the performance of these methods with Harrell's C‐index.

## 2 METHODS

### 2.1 Dataset

In this retrospective study, 268 patients who were operated for NSCLC between January 2008 and June 2018 in the Department of Thoracic Surgery, Faculty of Medicine, Ankara University were included in the study. The study was approved by the Ethical Committee of the Faculty of Medicine, Ankara University (no: I4‐240‐20, date: May 15, 2020). Written informed consent was obtained from all participants included in this study. Patients with Stage I pathological staging were included. Patients who received neoadjuvant therapy, had positive surgical margins, had a rare type of lung cancer, had synchronous–metachronous lung cancer, and had missing data were excluded from the study. Risk factors that may affect recurrence, such as pathological stage, histological type, number of sampled mediastinal lymph node stations, type of resection, and SUVmax of the lesion were recorded and included in the analysis. After the preprocessing step, the variables given in Table 1 were found to be both clinically important and statistically significant. In this study, models consisting of three (*X1*–*X3*), four (*X1*–*X4*), five (*X1*–*X5*), six (*X1*–*X6*), and seven (*X1*–*X7*) variables were built, respectively.

**TABLE 1:** Variables used in the data set sorted by clinical importance.

Variables	Description
*X1*	Pathological stage (Stage IA/IB)
*X2*	Sex (male/female)
*X3*	Age (years)
*X4*	Number of sampled mediastinal lymph node stations (≤2/>2)
*X5*	Histological type (Adenocarcinoma/squamous cell carcinoma)
*X6*	Resection type (wedge resection + segmentectomy/lobectomy + pneumonectomy)
*X7*	SUVmax of the lesion
Event	Recurrence (yes/no)
Time	Follow‐up (months)

### 2.2 Statistical analysis

The data were summarized as mean ± standard deviation and median (minimum–maximum) for continuous variables, and frequencies (percentiles) for categorical variables. Categorical variables were subjected to survival analyses using the Kaplan–Meier method, and the log‐rank test was employed to identify significant differences between groups. Multiple CoxPH model analysis was conducted to determine predictors of recurrence. Values of *p* < 0.05 were considered statistically significant. The primary aim of this study was to compare the classification performances of the DeepSurv, RSF, and CoxPH models in the NSCLC dataset. Statistical analyses were performed using Statistical Package for Social Sciences (SPSS, version 11.5). The dataset was randomly split into two mutually exclusive data sets, training and test sets. For all the models, including DeepSurv, RSF, and CoxPH the same training and test sets were utilized. For the RSF method, hyperparameter selection was performed using grid search. The number of trees was set to 1000 and different values for the number of nodes (3–25) and the number of randomly selected variables as candidates to split a node (mtry) (2–4) were tried. The best performance was achieved with 15 nodes, and mtry set to 3 (mtry = p/3 for regression, where p equals the number of variables). The hyperparameters used for DeepSurv were determined by the random search method to be optimal for the model and not to cause overfitting. The hyperparameters were used as follows: the number of epochs (2000), L2 regularization (0.01), dropout (0.20), the number of hidden layers (100), the number of nodes (10), learning rate (0.0001), learning rate decay (0.0001), momentum (0.90), and activation function (ReLU). DeepSurv, RSF, and CoxPH models were compared with Harrell's C‐index. Harrell's C‐index introduced in Harrell et al., 1982, is a widely used metric for evaluating risk models in survival analysis, especially when dealing with censored data.^12^ The C‐index is a measure related to the area under the ROC curve. It assesses the probability that, in a randomly selected pair of cases, the case that experiences an event earlier had a worse predicted outcome.^7^, ^12^ For each scenario, 100 training and test C‐index values of these three methods were recorded, and the prediction success of the methods was evaluated by the average of these values. The simulation of the data, RSF, and CoxPH models were performed with the R programming language (version. 3.6.3). “BiocManager,”^13^ “survcomp,”^14^ “survival,”^15^ and “dplyr”^16^ packages were used in the CoxPH analysis. The RSF method was implemented through the “randomForestSRC”^7^ package. Analysis of deep learning was carried out in the Python programming language. DeepSurv has been implemented in Theano^17^ with the “lasagne”^18^ package. A forest plot was also created with the help of the “forestmodel”^19^ package in the R programming language. For this study, the NC24 virtual machines from the NC series, equipped with NVIDIA Tesla K80 graphics cards and Intel Xeon E5‐2690 v3 (Haswell) processors, were utilized (vCPU: 24, memory: 224 GiB, SSD: 1440 GiB, GPU: 4, GPU memory: 48 GiB, max data disks: 64, max NICs: 4).

## 3 RESULTS

Of the 268 patients included in the study, 65 (24.3%) were female and the mean age was 61.69 ± 8.03 (27–83). Table 2 provides a summary of the general characteristics of the patients.

**TABLE 2:** The summary statistics of the patient characteristics.

Variables	Descriptive statistics
Sex, *n* (%)	
Male	203 (75.7)
Female	65 (24.3)
Age	
Mean ± SD	61.69 ± 8.03
Median (min–max)	62 (27–83)
SUVmax of the lesion	
Mean ± SD	8.36 ± 6.25
Median (min–max)	7.85 (0–32)
Resection type, *n* (%)	
Wedge resection + segmentectomy	50 (18.7)
Lobectomy + pneumonectomy	218 (81.3)
Histological type, *n* (%)	
Adenocarcinoma	184 (68.7)
Squamous cell carcinoma	84 (31.3)
Number of sampled MLNS, *n* (%)	
≤2	111 (41.4)
>2	157 (58.6)
Pathological stage, *n* (%)	
IA	149 (55.6)
IB	119 (44.4)

Abbreviations: Max, maximum; Min, minimum; MLNS, mediastinal lymph node stations; SD, standard deviation.

**FIGURE 1:**
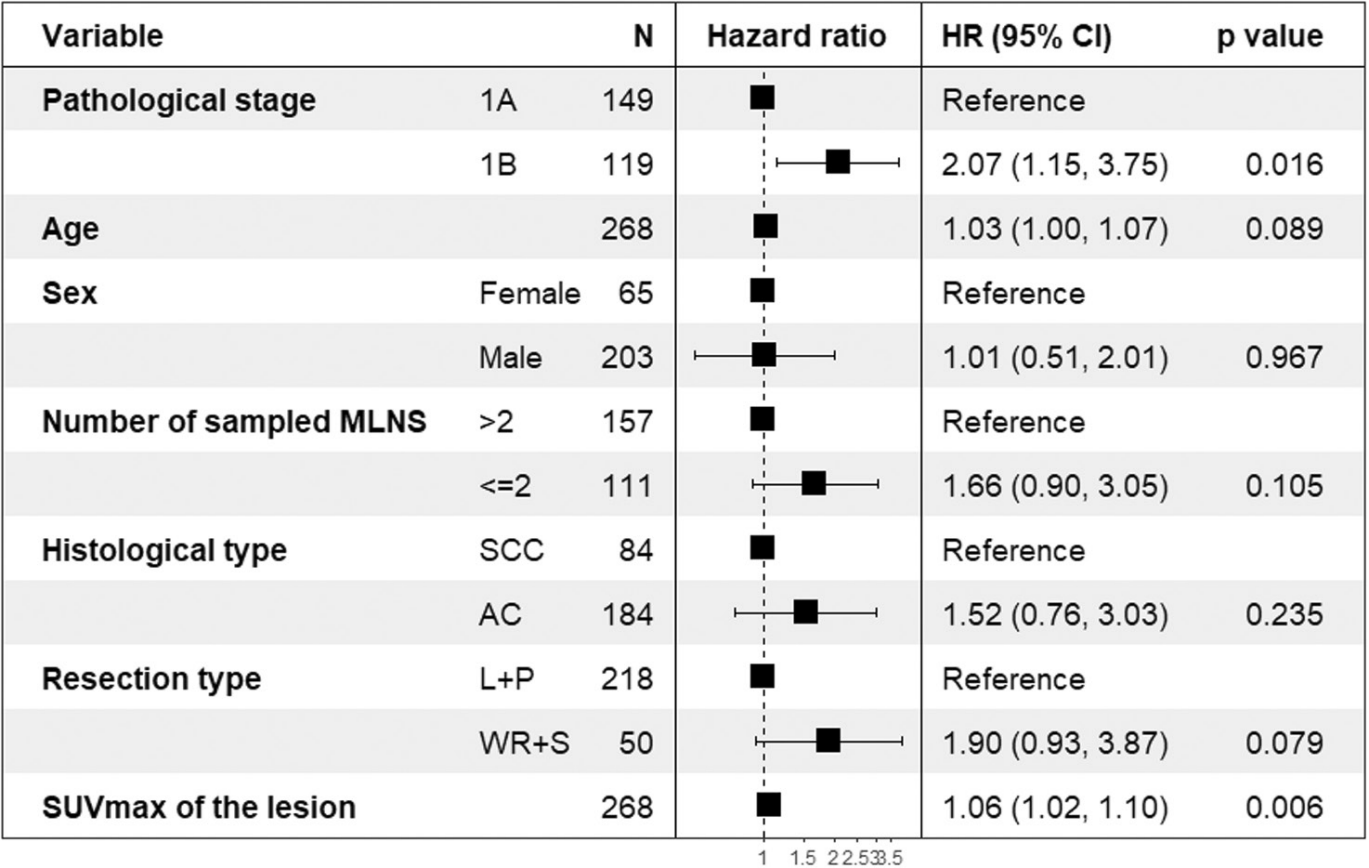
The forest plot showed the predictors of recurrence. AC: adenocarcinoma, CI: confidence interval, HR, hazard ratio; L + P, lobectomy + pneumonectomy; MLNS, mediastinal lymph node stations; SCC, squamous cell carcinoma; WR + S, wedge resection + segmentectomy.

Recurrence was observed in 52 (19.4%) patients. The 5‐year recurrence‐free survival rate of the patients was 74.7% ± 3.4. In the univariate analysis, a log‐rank test was conducted to assess the differences in survival distribution among the various types of pathological stage (*p* = 0.004), indicating a significant association with recurrence, while in multiple Cox regression analysis, pathological stage (*p* = 0.016), and SUVmax of the lesion (*p* = 0.006) were independent prognostic factors for recurrence (Table 3 and Figure 1).

**TABLE 3:** Univariate recurrence‐free survival (RFS) analysis results.

Variables	5‐year RFS (%)	*p*‐value
Sex		
Male	75.6 ± 3.7	0.849
Female	70.7 ± 8.2	
Age, HR [95% CI]	1.03 [1.00–1.07]	0.061
SUVmax of the lesion, HR [95% CI]	1.04 [1.00–1.07]	0.062
Resection type		
Wedge resection + segmentectomy	56.1 ± 11.1	0.108
Lobectomy + pneumonectomy	77.9 ± 3.4	
Histological type		
Adenocarcinoma	72.0 ± 4.3	0.270
Squamous cell carcinoma	80.8 ± 5.3	
Number of sampled MLNS		
≤2	64.4 ± 6.9	0.064
>2	79.9 ± 3.8	
Pathological stage		
IA	84.3 ± 3.7	0.004
IB	65.4 ± 5.4	

Abbreviations: CI, confidence interval; HR, hazard ratio; MLNS, mediastinal lymph node stations; RFS, recurrence‐free survival.

Starting with three variables that were incrementally added up to seven. The first three variables were clinically important variables, which should be included in the model according to expert opinion. Statistically insignificant and significant variables were subsequently added. Except for two variables—pathological stage and SUVmax of the lesion variables—although the five variables included in the study were statistically insignificant in the CoxPH analysis (Figure 1), they are still considered important in decision‐making and prognosis in the clinical setting.

**FIGURE 2:**
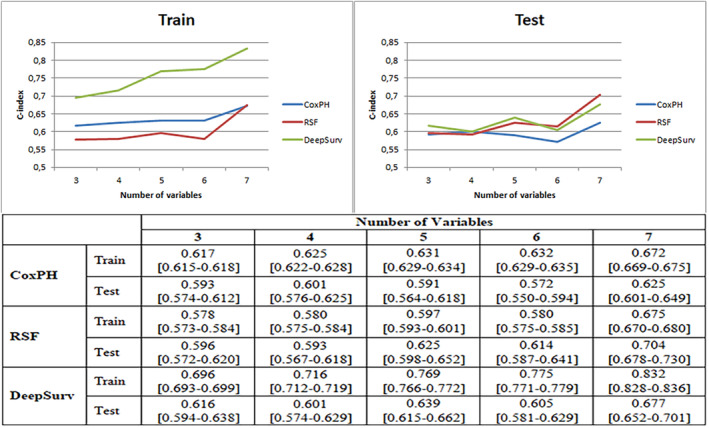
Performance of the DeepSurv, random survival forest (RSF), and Cox proportional hazard (CoxPH) model in terms of C‐index (95% confidence interval). 3: Age + Sex + Pathological Stage, 4: Age + Sex + Pathological Stage + Number of sampled MLNS, 5: Age + Sex + Pathological Stage + Number of sampled MLNS + Histological type, 6: Age + Sex + Pathological Stage + Number of sampled MLNS + Histological type + Resection type, 7: Age + Sex + Pathological Stage + Number of sampled MLNS + Histological type + Resection type + SUVmax of the lesion.

In the training set, as features were sequentially added, the C‐index of the DeepSurv and CoxPH methods exhibited a relatively steady upward trend. However, the RSF model showed a decrease in performance when the six‐feature model was added. Ultimately, DeepSurv demonstrated the highest performance among the three models in the training set, achieving a C‐index of 0.832, followed by RSF with a C‐index of 0.675, and CoxPH with a C‐index of 0.672. It was observed that the performance of DeepSurv increased with each newly added variable. The success of the DeepSurv method in training the model was found to be approximately 10–15% higher than the other two methods. In the test set, RSF demonstrated the highest performance among the three models, with a C‐index value of 0.704. It was followed by DeepSurv with a C‐index of 0.677, and CoxPH methods with 0.625 (Figure 2).

## 4 DISCUSSION

In survival analysis, classical statistical methods are generally used for low‐dimensional data, whereas machine learning‐based methods are used for high‐dimensional data. While analysis of survival data with classical statistical methods, such as CoxPH, has limiting assumptions such as proportionality of hazards, machine learning methods can overcome these limitations. In the healthcare field, interest in the use of machine learning in survival analysis has increased with the increasing size of datasets and the popularization of deep learning. There are some literature applying machine learning to lung cancer have reported good results. Yu et al.,^20^ employed a variety of machine learning algorithms, including naive Bayes, support vector machines (SVMs) with Gaussian kernel, SVM with linear kernel, SVM with polynomial kernel, bagging for classification trees, random forest using conditional inference trees, and Breiman's random forest. These algorithms were utilized to differentiate between lung cancer cases exhibiting distinct survival outcomes. Coudray et al,^21^ aimed to create a deep convolutional neural network designed for the accurate and automated classification of whole‐slide images sourced from the TCGA dataset. The objective was to distinguish between adenocarcinoma (LUAD), squamous cell carcinoma, and normal lung tissue. However, it is important to emphasize that the scope of this study was centered around survival analysis. She et al,^22^ constructed a deep learning survival neural network utilizing sequential cases of recently diagnosed Stages I–IV NSCLC. They examined 127 features encompassing patient attributes, tumor stage, and treatment approaches for analytical purposes. The performance evaluation of the models was conducted using the C‐index. The resultant deep learning survival neural network model demonstrated promising implications for both prognostic assessment and treatment guidance concerning lung cancer‐specific survival. While She et al,^22^ focused on a comprehensive analysis of consecutive cases involving newly diagnosed Stages I–IV NSCLC, our study specifically concentrated on patients within Stage I. In their recent study, Yu et al,^23^ acquired patient data concerning cutaneous malignant melanoma (CMM) from the Surveillance, Epidemiology, and End Results database. They employed DeepSurv model to forecast the survival rates of CMM patients and assess the model's efficacy. Their findings indicated that the DeepSurv model outperformed the CoxPH model in accurately predicting the survival time of individuals diagnosed with CMM.

In this study, the prognostic factors that may affect relapse in patients with operated Stage I NSCLC were analyzed using three different methods such as CoxPH, RSF, and DeepSurv, and compared the performance of these methods with Harrell's C‐index. Considering the findings from this study, it was found that all three methods had similar results. Here, our recommendation is to consider speed and cost. If speed is important, that is, if we want to obtain results quickly, a deep learning‐based survival prediction method using GPU may be preferred. Using GPUs may have another advantage to integrate DeepSurv to the decision support systems.

Careful measures were taken to assess and address the potential issue of overfitting in the DeepSurv model. Rigorous hyperparameter tuning and validation techniques were employed to ensure the model's ability to generalize well to unseen data. These steps aimed to enhance the reliability and robustness of the findings and minimize any biases introduced by overfitting.

A major strength of our study is the utilization of a well‐defined dataset comprising patients who underwent surgery for NSCLC. The use of mutually exclusive training and testing sets enhances the reliability of our model evaluation. Additionally, we employed rigorous hyperparameter tuning and validation techniques to optimize the performance of our models. The inclusion of clinically important variables highlights the relevance of our findings in a clinical setting.

However, several limitations need to be acknowledged. First, our study focused on a relatively small set of simple features, which may have limited the predictive power of the models. Incorporating additional complex features, such as genomic or molecular data, could potentially enhance the performance of the models. Second, our study relied on retrospective data, which may introduce biases. Future prospective studies with larger cohorts and diverse populations are needed to validate our results. Lastly, although we employed rigorous model evaluation techniques, external validation on independent datasets would further strengthen the reliability of our findings.

This study built the models and tested their performance with mutually exclusive training and testing on total 268 patients who were operated for NSCLC. A larger dataset from single center or multiple centers may improve these results and increase the performance of deep learning‐based survival prediction in patients with lung cancer. The findings align with existing literature and highlight the importance of feature selection, model optimization, and rigorous evaluation. While our study has certain limitations, it contributes to the growing body of knowledge in precision oncology and provides insights for further research and clinical application of machine learning in lung cancer prognosis.

## 5 CONCLUSION

This study provides novel insights into the application of machine learning models, including the pioneering integration of GPU, for predicting survival outcomes in Stage I NSCLC patients. Through a comprehensive evaluation, we compared DeepSurv, RSF, and CoxPH models and observed incremental improvement in DeepSurv's performance with the addition of more variables. However, RSF demonstrated the best performance in the test set. The clinical significance of the findings lies in the potential of machine learning models, particularly RSF, to assist clinicians in treatment decision‐making and tailored prognostic strategies. The results highlight the applicability of machine learning in precision oncology, offering valuable insights for patient prognosis and improving overall survival rates. The study acknowledges its limitations, such as the use of a small set of features and retrospective data, calling for future research to explore complex features and validate the findings in larger and diverse patient cohorts. In conclusion, the study demonstrates the potential of machine learning models, with GPU integration being one of the first examples, in predicting survival outcomes in NSCLC patients, providing a valuable approach for prognostic evaluation and treatment decisions in precision oncology.

## AUTHOR CONTRIBUTIONS

**İrem Kar:** Conceptualization (lead); formal analysis (lead); methodology (lead); software (lead); visualization (lead); writing – original draft (lead). **Gökhan Kocaman:** Conceptualization (supporting); data curation (equal); formal analysis (supporting); methodology (supporting); resources (equal); visualization (supporting). **Farrukh İbrahimov:** Conceptualization (supporting); data curation (supporting); investigation (supporting); resources (supporting). **Serkan Enön:** Data curation (supporting); resources (supporting); supervision (equal); writing – review and editing (equal). **Erdal Coşgun:** Conceptualization (equal); methodology (equal); software (equal); supervision (equal); writing – review and editing (equal). **Atilla Halil Elhan:** Conceptualization (equal); methodology (equal); supervision (equal); visualization (equal); writing – review and editing (equal).

## CONFLICT OF INTEREST STATEMENT

The authors declare no conflict of interest.

## Data Availability

The data that support the findings of this study are available on request from the corresponding author. The data are not publicly available due to privacy or ethical restrictions.
